# Trigeminal autonomic cephalalgias presenting in a multidisciplinary tertiary orofacial pain clinic

**DOI:** 10.1186/s10194-019-1019-7

**Published:** 2019-06-11

**Authors:** D. Y. Wei, D. Moreno-Ajona, T. Renton, P. J. Goadsby

**Affiliations:** 1 Headache Group, Department of Basic and Clinical Neuroscience, King’s College London, London, UK; 2 Oral Surgery, Institute of Dentistry, Kings College London, London, UK; 3 NIHR Wellcome Trust King’s Clinical Research Facility, SLaM Biomedical Research Centre, Wellcome Foundation Building, King’s College Hospital, London, SE5 9PJ UK

**Keywords:** Orofacial pain, Trigeminal autonomic cephalalgias, Hemicrania continua

## Abstract

**Abstract:**

Orofacial pain may have a variety of causes and offers a significant clinical challenge for its diagnosis and management.

**Objective:**

To assess the headache disorders presenting in a tertiary multidisciplinary orofacial pain clinic, after dental causes have been excluded.

**Methods:**

Clinic letters from the initial consultation and subsequent follow up reviews of the 142 patients, who were seen in the tertiary Multidisciplinary Orofacial Pain clinic between January 2015 until January 2018 were reviewed as a clinical audit.

**Results:**

The most common diagnoses were possible trigeminal autonomic cephalalgia (*n* = 62, 44%), migraine (*n* = 38, 27%) and painful post-traumatic trigeminal neuropathy (*n* = 17, 12%). The most common trigeminal autonomic cephalalgia diagnosis was hemicrania continua (*n* = 13, 9%), which is higher than the reported prevalence in neurology and headache clinics.

**Conclusion:**

This study demonstrates the importance of a multidisciplinary approach to diagnosing complex orofacial pain patients and the importance of awareness of primary headache disorders, in particular trigeminal autonomic cephalalgias, thereby reducing unnecessary diagnostic delays or procedures.

## Introduction

Trigeminal autonomic cephalalgias (TACs) are characterized by unilateral intense pain involving predominantly the orbito-temporal region and associated with presence of ipsilateral cranial autonomic symptoms [1]. At least one or more should be present among conjunctival injection, lacrimation, nasal congestion, rhinorrhoea, eyelid oedema, forehead and facial sweating, miosis and/or ptosis [2].

The distinction between the four different TAC conditions is based on headache duration, frequency and treatment response. Namely, cluster headache (CH) attacks last 15–180 min when untreated, with a frequency of up to 8 attacks a day. Paroxysmal hemicrania (PH) attacks are shorter in duration (2–30 min) and usually more frequent reaching a maximum of 40 attacks per day. Short-lasting Unilateral Neuralgiform headache attacks with Conjunctival injection and Tearing (SUNCT), and Short-lasting Unilateral Neuralgiform headache Attacks with cranial autonomic features (SUNA) are the shortest type of TAC (1 s-10 min) [2] and may occur up to 100 times daily. Hemicrania continua (HC) patients have a background of continuous unilateral pain with attacks of worsenings. PH and HC, respond specifically to therapeutic treatment with indomethacin [2, 3].

The incidence of TACs is low, with CH being the most common with a prevalence of approximately 0.1% of the population [4].

The pathophysiology of these entities is thought to involve the hypothalamus in its posterior, lateral and paraventricular nucleus, the trigeminovascular complex and the parasympathetic fibres with a crucial role of the sphenopalatine ganglion via the trigeminal autonomic reflex [5].

From neuroimaging studies, the acute phase of pain involves the ipsilateral hypothalamus in CH [6], the contralateral in PH [7] and HC [8], and the ipsilateral [9] and bilateral in SUNCT [10].

The pain CH patients experience can be easily and often misdiagnosed as dental pulp pain. Interestingly, Peñarrocha and colleagues explored the possible relationship between oral surgery, endodontic procedures and CH. The authors described a series of 54 patients who underwent oral surgery and endodontic procedures [11]. Among them, 58% had a dental procedure ipsilateral to the side where CH developed and in 24 out of 54 (44%) oral surgery was performed afterwards in order to resolve this pain. Although the authors hypothesized a possible relation between nerve damage and subsequent development of CH, they also admitted that dental extraction and endodontics could have been performed in response to CH-related pain. However, the authors did not include information about the presence of paraesthesia, dysesthesia, or allodynia which has been described in patients suffering from neuropathic pain after invasive dental procedures [12].

With regards to patients with CH, Bahra and Goadsby documented that dentists and ear, nose and throat (ENT) surgeons were most commonly consulted prior to neurologists (45% of 511 subjects) and that 52% had an invasive procedure performed for pain [13]. The authors found the mean time to diagnosis of CH in the 1990s was 2.6 years compared to in the 1950s which was 22.3 years [14].

The application of the ICHD-II criteria to 502 patients who presented with Temporo-Mandibular Dysfunction (TMD) and orofacial pain in a Temporo-Mandibular Joint and Orofacial Pain Clinic found, surprisingly, one single case of CH with no other TACs reported, and being tension-type headache the most frequent entity, diagnosed in 246 patients [14].

From a neurologist’s perspective, a prospective study of all surgical referrals to one consultant neurologist in the UK over a 42-month period found 12 patients from oral and maxillofacial surgery clinics being 5 diagnosed of trigeminal neuralgia and 3 of atypical facial pain [15]. Most recently, a systematic review of diagnostic and therapeutic errors in TACs performed by Viana and colleagues [16] showed that patients with CH were found to be the largest category of mismanaged patients. Few case series included mismanagement of HC [17, 18]. Rossi and colleagues described 25 patients fulfilling the ICHD-II criteria for HC among 1612 subjects attending an Italian Headache Centre over a three-year period [18]. Among these patients, 52% had previously been misdiagnosed with migraine. Only two published cases of misdiagnosed SUNCT appeared in the review, both were incorrectly classified as Trigeminal Neuralgia (TN) [16].

This study aims to evaluate the headache disorder diagnoses seen at a tertiary Multidisciplinary Orofacial Pain (OFP) clinic and more specifically the diagnoses and management of TACs. The work has been presented in preliminary form at the Congress of the International Headache Society (Vancouver, Canada, 7–10 September, 2017) [19].

## Methods

Clinic letters from the initial consultation and subsequent follow up reviews of the 142 patients, who were seen in the tertiary Multidisciplinary Orofacial Pain clinic between January 2015 until January 2018 were reviewed as a clinical audit. In this clinic, all patients are first reviewed by an experienced Orofacial Surgeon (TR) to exclude a dental cause for their facial pain. The Multidisciplinary Orofacial Pain clinic has approximately 300 new patients a year, approximately 12% of patients present with a dental cause for primary complaint, of which the most common is painful post-traumatic trigeminal neuropathic (48%) and TMD is second most common (23%). Patients are then seen by Headache fellows under the supervision of a Headache Specialist (PJG). For these patients, their demographics, the presence of dental work, time until diagnosis and diagnosis were recorded. Of the patients with a possible TAC diagnosis, the pain localisation, cranial autonomic symptoms and result of the indomethacin test (if applicable) were recorded. The diagnoses were made according to ICHD-III beta [20].

### Indomethacin response

Indomethacin response was determined either by intramuscular indomethacin placebo-controlled testing with 150 mg indomethacin or by course of oral indomethacin, with a titrating regime starting at 25 mg three times a day in the first week, increased to 50 mg three times a day for a week and finally 75 mg three times a day. A positive test was deemed to be at least a 50% reduction in pain intensity and/or 50% in frequency or duration of the worsenings present with the indomethacin and not with the placebo.

### Analysis

Data were collected in Excel, where quantitative analysis was performed, and STATA (SE 15.1 for Mac) was used for chi square and Fischer’s exact testing..

## Results

**Fig. 1:**
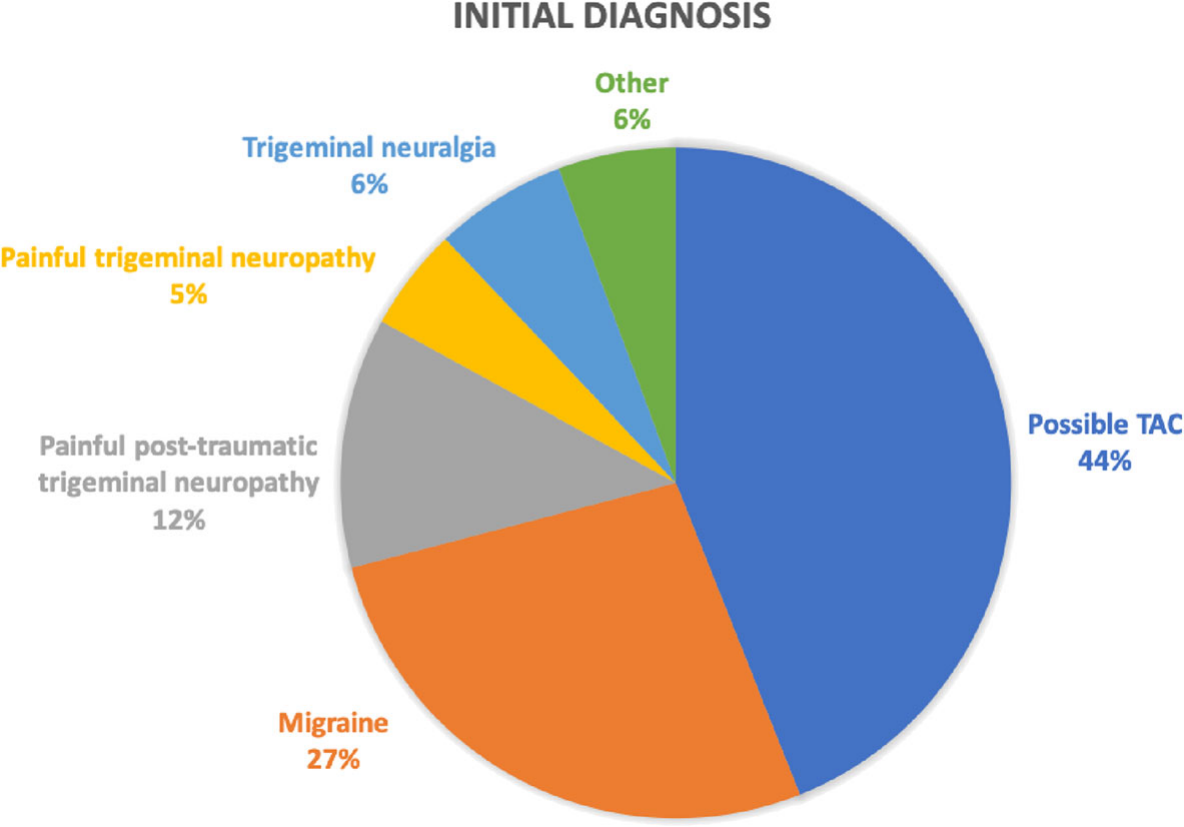
Initial diagnosis following consultation

Of the one hundred and forty-two patients reviewed; there were 100 women (70%) and 42 men (30%). The mean age was 51 ± 3 (52 ± 4 for men, 50 ± 3 for women, mean ± SD). Among all consultations, 109 (77%) were new patients and 33 (23%) were follow ups. A TAC was suspected in 62 patients (44%) based on the history of unilateral pain in the trigeminal distribution and the presence of ipsilateral cranial autonomic symptoms, as per ICHD-III beta criteria. Of the remaining patients, the most common diagnoses were chronic and episodic migraine, painful post-traumatic trigeminal neuropathy and trigeminal neuralgia (Fig. 1 and Table 1). There was one patient that was initially diagnosed as intermedius nerve neuralgia following the first consultation, however, with subsequent review was diagnosed with SUNA and is included into the TAC cohort. One patient was referred with dystonia and therefore excluded from the cohort. Interestingly, 18 patients were diagnosed with more than one headache type (Table 2), the most common presentation being the presence of painful post-traumatic trigeminal neuropathy and migraine.

**Table 1:** Initial diagnosis of patients in the cohort, taking into account that some of them had more than one headache type (Table 2); CSF: Cerebrospinal fluid; ^a^One patient excluded, was diagnosed with dystonia

Initial diagnosis^a^	Number of patients (%)
Possible TAC	62 (44%)
Migraine	38 (27%)
Chronic migraine	27 (19%)
Episodic migraine	11 (8%)
Painful post-traumatic trigeminal neuropathy	17 (12%)
Painful trigeminal neuropathy	7 (5%)
Trigeminal neuralgia	9 (6%)
Other:	9 (6%)
Nummular headache	2 (1.5%)
Primary stabbing headache	2 (1.5%)
Hypnic headache	1 (0.5%)
Burning mouth syndrome	1 (0.5%)
Headache attributed to low CSF pressure	1 (0.5%)
Posterior circulation stroke	1 (0.5%)
Intermedius nerve neuralgia	1 (0.5%)

**Table 2:** Patients with two headache diagnoses: Short-lasting Unilateral Neuralgiform headache attacks with Conjunctival injection and Tearing (SUNCT), and Short-lasting Unilateral Neuralgiform headache attacks with cranial Autonomic features (SUNA)

Headache types	Number of patients/ total with condition (%)
Migraine *and*	
Painful post-traumatic trigeminal neuropathy	8/17 (47%)
Trigeminal neuralgia	2/9 (22%)
Primary stabbing headache	1/2 (50%)
Painful trigeminal neuropathy	1/7 (14%)
Headache attributed to low CSF pressure	1/1 (100%)
Myofascial pain	1/1 (100%)
Hemicrania continua *and*	
SUNCT/ SUNA	2/6 (33%)
Primary stabbing headache	1/2 (50%)
Painful post-traumatic trigeminal neuropathy *and*	
Primary stabbing headache	1/2 (50%)

### Diagnosis prior to clinic

A previous suspected diagnosis was only reported in 17 patients, including trigeminal neuralgia (*n* = 6), migraine (*n* = 8), SUNCT (*n* = 1) and painful trigeminal neuropathy (*n =* 2). However, only 7 had a correct previous diagnosis and, interestingly, trigeminal neuralgia was the most commonly misdiagnosed (5/6 patients). As a previous comorbidity, temporo-mandibular dysfunction, was present in 10 (7%) patients. Neuroimaging (either MRI or CT) had been performed in 72 patients (51%) by the time they attended the consultation.

### Delay to diagnosis

Time to diagnosis for the whole cohort was 5.6 years, in patients with a confirmed TAC (HC, PH, CH and SUNCT/SUNA) was 7 years. Time to diagnosis in patients with HC, the most common diagnosis among TACs, was 8.7 years. In the two CH patients the delay was 6 and 17 years, in the one case with chronic PH the delay was 12 years and in SUNCT/SUNA patients (*n* = 6) the average delay was 5 years.

### TAC patients

TAC was suspected in 62 patients. This group included 40 women (65%) and 22 men (31%). The mean age was 53 ± 4 years (54 ± 6 for men, 53 ± 4 for women).

This group comprised of 13 confirmed HC, 1 confirmed PH, 6 SUNCT/SUNA, 2 CH, 17 negative indomethacin (chronic migraine), 21 possible HC and 2 possible PH (not confirmed) patients (Table 3). In the group with possible HC and possible PH, the clinical phenotype fits the ICHD-III beta, however had equivocal indomethacin response or from patient’s personal choice did not proceed with the indomethacin test.

**Table 3:** TAC diagnosis

Headache type within the possible TAC group	Number of patients (%)
Cluster headache	2/62 (3%)
Confirmed hemicrania continua (Positive Indotest)	13/62 (21%)
Confirmed paroxysmal hemicrania (Positive Indotest)	1/62 (2%)
Possible hemicrania continua^a^	21/62 (34%)
Possible paroxysmal hemicrania^a^	2/62 (3%)
Chronic migraine (negative Indotest)	17/62 (27%)
SUNCT/ SUNA	6/62 (10%)

^a^Clinical phenotype with equivocal indomethacin response or patients who did not proceed with the Indotest

### Pain location and description

As expected from a population of patients undergoing OFP assessment, most patients referred their pain to the second and/or third trigeminal branches (60/62), being only two cases of strictly V1 pain. Pain located in V2 (18/62) as well as V2 and V3 (17/62) were the most commonly reported followed by pain in V1 and V2 (9/62), hemifacial pain involving all three branches (13/62) and pain exclusively on V3 (3/62).

Description of the pain varied widely among patients. However, the pain was described, at least at times, as throbbing by 25 out of 62 patients. This type of pain, was present in migraineurs and non-migraineurs, although it was significantly associated with a migrainous background (Chi square: *p* − 0.001) but not with a negative indomethacin test (Fisher’s exact, *p* = 0.157). Pain was also described as pressure-like, stabbing, sharp, shooting, dull, burning or shooting/electrical, with no noteworthy differences among patients. Pain was rated as moderate to severe in all cases.

### Cranial autonomic symptoms

Of the patients with a possible TAC diagnosis, the average number of cranial autonomic symptoms per patient was 3. Patients with a confirmed TAC diagnosis (*n* = 22) reported on average 2.5 cranial autonomic symptoms per patient. The most common cranial autonomic symptom, within the whole group with possible TAC, was lacrimation (*n* = 35), followed by nasal congestion (*n* = 27) and conjunctival injection (*n* = 23).

### Oral procedures in the possible-TAC-population

Specifically, among patients with possible TAC (including all patients with suspected HC who underwent the indomethacin test), different dental procedures were recorded. Overall, dental extraction was the most common (15/62; 24%), followed by root canal treatment (8/62; 13%) and dental filling (4/62; 6%). Dental implant, minor trauma in the gum, mucosal biopsy and parotid surgery were performed in single cases (Table 4).

**Table 4:** Dental procedures in possible TAC patients

Oral procedure	Number of patients (%)
Dental extraction	15/62 (24%)
Root canal treatment	8/62 (13%)
Dental filling	4/62 (6%)
Dental implant	1/62 (2%)
Mucosal biopsy	1/62 (2%)
Parotid surgery	1/62 (2%)
Oral minor trauma	1/62 (2%)

A significant association (χ^2^ = 26.2; *p* = 0.000) was found between the variables “previous oral work” (25/62) and the variable “pain within 1 week after the procedure” (16/62), however this test does not show causation and there is a potential selection bias as all the patients have been referred from dentists and oral surgeons. Additionally, 10 patients underwent dental work afterwards to treat their pain, with only one patient getting some pain relief. A single patient underwent 3 procedures (tooth extractions) with no benefit. Only one procedure to treat the pain was performed in the remaining patients.

### History of headache and pain syndromes

Twenty-six out of 62 patients (42%) had a previous pain background, migraine being the most common. Namely, 16 patients had been previously diagnosed with episodic migraine without aura, 5 with episodic migraine with aura and 2 with chronic migraine. Two patients had fibromyalgia and one patient had temporomandibular joint dysfunction and bruxism.

### Indomethacin test

An indomethacin test was performed in 31 patients (26 intramuscular and 5 oral).

After the administration of the drug, pain relief was observed in 14 (positive) patients, with no benefit in the remaining 17 subjects (negative).

## Discussion

TACs often present to other specialities given the distribution and location of the pain. Patients often see dentists and orofacial specialists to find the root of the pain. Therefore, a multi-disciplinary team approach involving dental, oral surgery team and headache specialists is important to optimise patient outcomes. The patients presenting to our Multidisciplinary Orofacial Pain clinic are often complex and have had dental causes excluded as the reason for the pain. Within our cohort, the most common initial diagnosis was possible TAC, this was more common than migraine. Most patients with possible TAC, localised their pain to the second and/or third trigeminal nerve distribution, this should be considered with some care and can be explained by selection bias as most patients presented to a dentist for the pain initially.

The delay to diagnosis for patients with a TAC (HC, PH, CH and SUNCT/SUNA) was 7 years, in particular the delay until diagnosis for CH was 6 years for an episodic CH patient and 17 years for a chronic CH patient; this is higher than the average delays reported in a systemic review [16] and case series, where the range was 2.6 years to 11 years [13]. Although the delay to diagnosis for CH in this study is longer, it is not a true representation of the time to CH diagnosis, as there were only two cases in this cohort and it is likely that with the education of many dentists, CH cases are diagnosed earlier and therefore not presenting in a tertiary Orofacial Pain clinic. There was only one case of PH, and the delay until diagnosis was 12 years, this is within the range reported in the systemic review (range 10 months-12 years) [16]. The average delay till diagnosis was 5 years. The pooled mean delay of diagnosis in HC has been reported to be 8 ± 7.2 years [16], this is similar to what we have found, a delay of 8.7 years. TACs are generally not recognised and therefore diagnosis is often delayed, furthermore there is a large group of TAC patients that present to the orofacial services.

Traditionally, HC has been considered a rare condition and a recent study by Hryvenko and colleagues reviewed 1617 new patients seen in their TMD and Orofacial Pain Clinic, and only found 6 (0.4%) patients with HC, with 5 patients with nearly complete response with oral indomethacin (150-225 mg/day) and one patient had to remain on 75 mg/day due to intolerable side effects, and therefore had partial pain relief [21]. In a pooled analysis of published HC case series, HC represents 1.7% (range 1.3–2.3%) of neurology or headache clinics patients [22]. We have found a higher prevalence of HC in our cohort of 142 patients from our tertiary Multidisciplinary Orofacial Pain clinic. There were 13 patients (9%) with confirmed HC on either placebo-controlled intramuscular indomethacin or oral indomethacin titrating trial. Our findings suggest HC patients present to orofacial pain services due to the distribution and characteristics of the pain, and we encourage orofacial pain services to consider this diagnosis perhaps more often than may have been the case. SUNCT/SUNA was the second most common TAC diagnosis made in our cohort, 6 patients (10%).

### Limitations

This is a retrospective study of clinic letters to identify patients who attended the Multidisciplinary Orofacial Pain clinic, therefore we cannot assess any predictive or causative factors. The clinical documentation is, however, structured and systematic since a priori we have seen patients referred from such services before our new service was initiated. A strength of the study is the certainty of the diagnosis, as we followed the patients carefully through the years. Importantly, the patients with HC were confirmed with placebo-controlled intramuscular indomethacin testing.

## Conclusion

TACs are an important group of primary headache disorders dentists and oral surgeons should be aware of as many patients with unilateral side-locked primary headaches present to the dental services. Neurologists should be cognisant of this presentation pattern when seeing patients from these pathways. HC is underdiagnosed and this may be due to the presentation to other specialities rather than to neurology and headache services. We have found the most effective way to ensure patients are diagnosed correctly and managed optimally is a multidisciplinary service with dentists, oral surgeons and headache specialists.

## Data Availability

All data generated or analysed during this study are included in this published article.

## Abbreviations

CH: Cluster headache
PH: Paroxysmal hemicrania
SUNA: Short-lasting Unilateral Neuralgiform headache attacks with cranial Autonomic features
SUNCT: Short-lasting Unilateral Neuralgiform headache attacks with Conjunctival injection and Tearing
TAC: Trigeminal autonomic cephalalgia
TN: Trigeminal neuralgia
